# The impact of nasal aspiration with an automatic device on upper and lower respiratory symptoms in wheezing children: a pilot case-control study

**DOI:** 10.1186/s13052-018-0489-6

**Published:** 2018-06-14

**Authors:** Antonio Pizzulli, Serena Perna, Anja Bennewiz, Holger Roeblitz, Salvatore Tripodi, Jakob Florack, Petra Wagner, Stephanie Hofmaier, Paolo Maria Matricardi

**Affiliations:** 1 Practice for Pediatric Allergy and Pneumology, Berlin, Germany; 2 Department of Pediatric Pulmonology, Immunology and Intensive Care Medicine, Charité - University Medicine Berlin, Augustenburger Platz, 1, 13353 Berlin, Germany; 3 Practice for General Pediatrics and Pediatric Cardiology, Berlin, Germany; 4 Practice for General Pediatrics and Pediatric Allergy, Berlin, Germany; 5 Pediatric Department and Pediatric Allergology Unit, Sandro Pertini Hospital, Rome, Italy

**Keywords:** URI, LRI, Childhood, Therapy, Prevention, Nasal aspiration, Nebulizer, Salbutamol

## Abstract

**Background:**

The impact of proper aspiration of nasal secretions during upper respiratory infection on the frequency and severity of symptoms of lower airways has never been investigated. The study was aimed at testing if cleaning the nasal cavities of children with recurrent wheezing using an automatic nasal aspirator improves the upper and lower respiratory symptoms during the cold season.

**Methods:**

Parents of wheezing children (age 3-72 mo.) answered questionnaires and learned using a nebulizer equipped (cases) or not equipped (controls) with an automatic nasal aspirator (DuoBaby, OMRON, Japan). During a 90-days monitoring period parents filled an electronic diary (BreathMonitor, TPS, Rome, Italy) on their child’s symptoms of the upper and lower airways.

**Results:**

Eighty-nine/91 patients (43 cases, 46 controls) completed the study. Less days with upper (25.0% vs 46.4%, *p* = 0.004) or lower (21.8% vs 32.8%, *p* = 0.022) airways symptoms and less days with salbutamol inhalation (12.2% vs 16.9%, *p* < 0.001) were reported by cases than by controls. The episodes of upper respiratory symptoms were shorter [4.3 days (95%CI:3.8–4.9) vs 5.7 days (95%CI:5.0–6.4), *p* = 0.007] but not less frequent [2.3 (95%CI: 1.8–2.8) vs 2.6 (95%CI:2.2–3.0), *p* = 0.122] among cases than among controls. Similarly, the episodes of lower respiratory symptoms tended to be shorter [3.8 days, (95%CI: 3.4–4.2) vs 4.4 days, (95%CI: 4.4–6.0), *p* = 0.067] but not less frequent [1.9 (95%CI:1.5–2.3) vs 2.1 (95%CI:1.7–2.4), *p* = 0.240] among the group using the nasal aspirator.

**Conclusions:**

In our pilot study, the use of an automatic nasal aspirator in children with a history of recurrent wheezing was associated with an improved respiratory health during the cold season.

**Electronic supplementary material:**

The online version of this article (10.1186/s13052-018-0489-6) contains supplementary material, which is available to authorized users.

## Background

Infections of the upper and lower airways as well as wheezing are the first causes of doctor’s consultation in the first 2 years of life. Their social and economic burden at a worldwide level is enormous [1, 2]. First-line treatment of airways infections is frequently based on local, rather than systemic drugs. Local treatments include corticosteroids and β2-agonists for the lower airways as well as corticosteroids, nasal anticholinergics and decongestants for the upper airways [3, 4]. Drugs can be administered with metered dose inhalers (MDI), nebulizers, or other devices [5, 6]. Young families, predominantly in the western societies and especially in their first born child, are not well trained to face the normal consequences of respiratory infections in their child [7]. Thus, upper respiratory infections (URI) and lower respiratory infections (LRI) result often in unnecessary frequent doctor’s consultations, work days lost and over-treatment with antibiotics, etc. [7, 8].

Upper and lower respiratory infections often share etiologic and risk factors, they are pathophysiologically linked and appear frequently associated [9, 10], leading to the concept of “United Airway Disease” (UAD) [11–13]. Under the UAD concept, prompt and proper treatment of URI is considered beneficial to reduce the burden of LRI. In particular, maintaining the nasal cavity “free” from mucus (aspiration, lavage) is an additional advice given by most pediatricians to treat URIs. However, this advice is normally not heeded by parents and it is not known whether this intervention is able to improve the child’s respiratory health.

The study was aimed at testing the working hypothesis that proper cleaning of the nasal cavities of children with recurrent wheezing, using an automatic nasal aspirator, improves the upper and lower respiratory symptoms and reduces the use of medication during the cold season. To this end, we monitored the daily symptoms and use of beta-2 adrenergic inhalation (salbutamol) of randomized pre-school children with a history of wheezing. During the 3 months monitoring period, patients included in the case group received a nebulizer equipped with an automatic nasal aspirator to use at their homes, while the control group received the same nebulizer without the nasal aspirator.

## Methods

### Study population and design

The “Breathe-Free-Baby” study is a case-control study on the impact of device-assisted nasal aspiration on respiratory health. The study is structured in a time “0” (T0) visit, a monitoring period, and a time “1” (T1) visit. Children seeking care in the participating clinical centers for symptoms of the upper and/or lower airways between December 2016 and February 2017 were examined as candidates for enrolment. Criteria for eligibility were: 1) one or more episodes of doctor’s diagnosed wheezing and/or recurrent cough requiring salbutamol inhalation occurring in the last 12 months; 2) age between 3 and 72 months; 3) sufficient comprehension of the German language; 4) availability of a smart-phone and/or a personal computer with ADSL connection at home. Exclusion criteria were: 1) anatomic malformation causing chronic nasal and/or bronchial obstruction; 2) severe chronic disease; 3) contraindication for the use of beta sympathomimetic drugs; 4) intention to move away from Berlin during the monitoring period. Recruited patients were further randomized and allocated to one of the two groups: cases receiving the nebulizer with nasal aspirator and controls receiving the nebulizer without nasal aspirator. The study design included a first visit (T0), followed by a monitoring period and by a final visit (T1).

### T0 visit

During the first study visit (T0), the doctor informed the parents in detail about the study protocol and procedures. Parents answered a list of questions on sociodemographic information and symptoms. The study nurse instructed the parents about the set-up, use and cleaning of the nebulizer. Cases were also instructed to use the nasal aspirator. All parents were asked to test their capability to properly use the DuoBaby nebulizer for the inhalation of Salbutamol Ampoules in case of cough or wheezing and (cases only) its nasal aspirator in the office under the nurse’s supervision. Parents were asked to use the nasal aspirator with an “as needed” approach, i.e. in the presence of nasal symptoms (e.g. blocked nose, rhinorrhea, sneezing, etc.). Parents were instructed by the study nurse to properly check whether the nose cavities were free of mucus and when the use of the nasal aspirator had to start/finish. The mobile application (App) for the electronic diary “BreathMonitor” (TPS, Rome, Italy) was downloaded and installed on the parents’ smart phone. The App’s proper use was explained to the parents.

### Monitoring period

The day-by-day data on symptoms and medication use, as well as data on the quality of life, were recorded via the electronic diary (e-Diary) (BreathMonitor®, TPS, Rome, Italy) by the patients’ parents during the monitoring period. Parents were asked to start filling the e-Diary on the day following the T0 visit (Additional file 1: Table S1). The monitoring period was of 90 days or more. The patients were able to consult the doctors and their co-workers as usual according to routine procedures. The “BreathMonitor” App included questions on upper and lower airways symptoms, their triggers and impact on daily life, as well as the use of daily medication. Hospitalization, doctor or nurse consultations were also registered.

### T1 visit

At the end of the monitoring period each child was examined again (T1 visit). The parents filled the three questionnaires on: A) symptoms and quality of life related to the child’s respiratory health in the monitoring period; B) use of the DuoBaby nebulizer unit (usability, acceptance, efficacy, etc.) (Additional file 1: Table S2); C) use of the DuoBaby nasal aspirator unit (only cases) (usability, acceptance, efficacy, etc.) (Additional file 1: Table S3).

### The DuoBaby device

The “DuoBaby” nebulizer (OMRON Healthcare, Kyoto, Japan) has been thoroughly tested and certified by the European Community for its standard use in children (Additional file 1: Figure S1). The device is equipped with two different nebulization tops, of which one is specifically designed for treating the upper airways (MMAD approximately 9 um) and one for treating the lower airways (MMAD approximately 4 um). In addition, DuoBaby contains a nasal aspirator which, thanks to the venturi effect, creates a vacuum with a suction force between 0.15 and 0.2 bar. The produced suction allows mucus to be removed from the nasal cavities and is collected in a collection chamber.

### Randomization

Randomization was performed by allocating each patient with a 1:1 ratio between treatment and control group according to a simple randomization list prepared by an external researcher using R statistical software (R Core Team, 2014), version 3.2.3. Each name of the allocation sequence was put in sequentially numbered, opaque, sealed envelopes and opened by the Doctor at the T0 visit only after the participant’s name was written on the appropriate envelope. The Doctor registered the allocation and then communicated it to the patient. The allocation group of each patient was blinded to the statistician who analyzed data.

### Primary and secondary outcome

The study was aimed at testing the working hypothesis that regular and proper cleaning of the nasal cavities of children with recurrent wheezing, using an automatic nasal aspirator, improved the upper and lower respiratory symptoms.

As secondary objectives the study tested if regular assisted aspiration improved aspects of the family’s quality of life linked to the child’s respiratory health and reduced the use of medication during the cold season. Moreover the degree of usability, acceptability and tolerability of the nebulizer (case and controls) and of the aspirator (cases) devices was tested.

### Sample size

Mean frequency of doctor’s consultations and/or hospitalization due to wheezing and/or recurrent cough during the “cold” season (January–April) in the category of eligible children is in the study settings (offices of Dr. Pizzulli and colleagues) around 1.5 per month. This corresponds to 6 episodes in the observation period of four months (≈ 120 days). We considered clinically relevant at least a 12% reduction of this rate (i.e. from 6 to 5.3 episodes with a standard deviation equals to 1) in the group of children using DuoBaby nasal aspirator (cases). Considering an alpha error of 5% and a power of 90%, 43 subjects per each group were needed. Considering a 15% drop out 50 patients per group were recruited.

### Statistical analysis

Data were summarized as numbers (n) and frequencies (%) if they were categorical and as mean/median and standard deviation (SD)/interquartile range (IQR) if quantitative. Chi-squared test, when conditions were respected, or Fisher test was used to evaluate the association of categorical data between groups. Mann Whitney U test was used to compare quantitative not normally distributed variables between groups. To assess the normal distribution of quantitative data, the Shapiro–Wilk test was applied. The severity of respiratory symptoms during the monitoring period was calculated at individual level on the basis of the data collected with the e-Diary. At group level, for each parameter, the cumulative number of days with each symptom recorded by all the cases or controls was referred to the cumulative number of reported days by all the cases or controls, respectively. At individual level, for each parameter, the percentage of days with symptoms was referred to as the total of the recorded days by the patient. We defined an episode of upper or lower respiratory symptoms as the period of time (number of days) with symptoms between the last day without symptoms and the last recorded day with symptoms. Multilevel mixed-effects were applied to take into account repeated measures of the same patients for analysis on episode’s duration. A *p*-value < 0.05 was considered statistically significant. Statistical analyses were performed with R statistical software (R Core Team, 2014), version 3.2.3.

## Results

### Characteristics of the study population

**Fig. 1:**
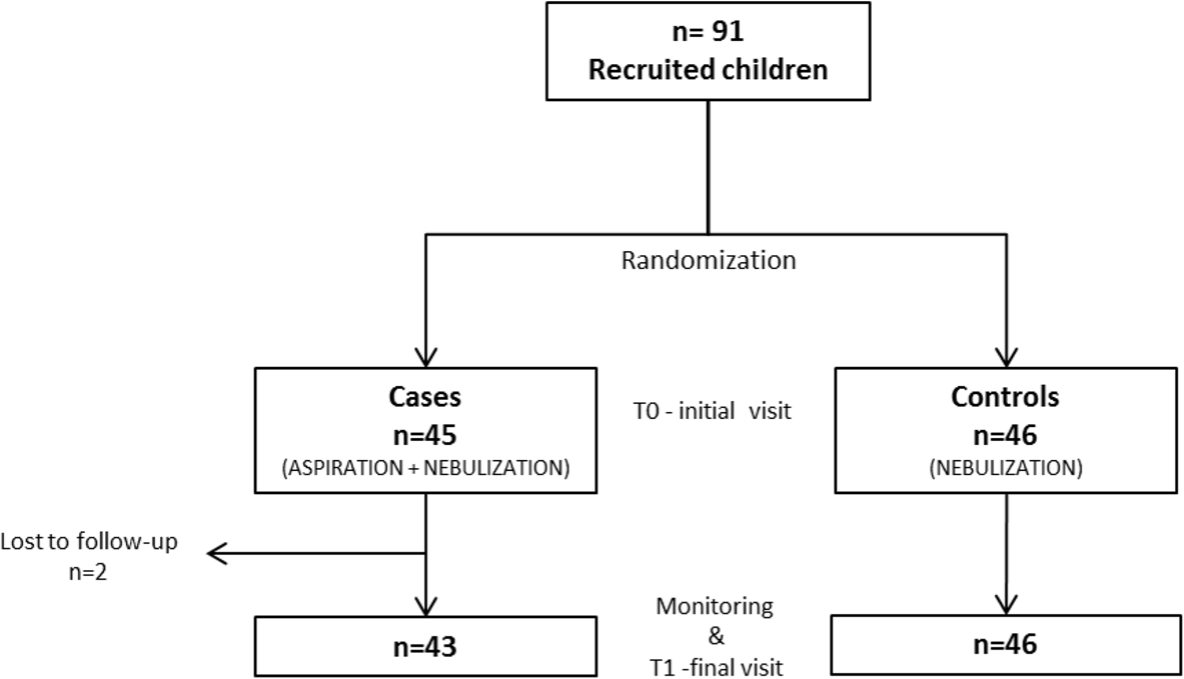
Flow diagram showing the population sampling

Ninety-one patients with symptoms of the upper and/or lower airways met the inclusion criteria and were 1:1 randomly assigned to the cases group receiving the nebulizer with nasal aspirator (*n* = 45) or the controls receiving the nebulizer without nasal aspirator (*n* = 46). Two patients in the group receiving the nebulizer plus nasal aspirator did not complete the monitoring period and were excluded from the analyses (Fig. 1). Sixty one (69%) of the participants were male and their average (SD) age in months was 32.5 (20.5). The majority of them were Caucasians (76%) and mothers answered to the questionnaire more frequently (64%). No significant differences between the two study groups were observed in one or more events of cough outside cold and in the frequency of indirect markers of asthma severity: sleep disorders, emergency events, nights in the hospital and days missed at pre-school/daycare (Table 1).

**Table 1:** Characteristics of the patients using the nebulizer equipped (cases, *n* = 43) or not equipped (controls; *n* = 46) with nasal aspirator

	Cases		Controls		
	*n* = 43^b^		*n* = 46^b^		p^a^
Male gender (n; %)	27	*62.8*	34	*73.9*	0.361
Age (months) (median, IQR)	30	*(20–60)*	35	*(18–52)*	0.767
Older sibling (n; %)	18	*41.9*	23	*50.0*	0.441
Younger sibling (n; %)	4	*9.3*	8	*17.4*	0.356
Full breast-feeding (4 month) (n; %)	33	*76.7*	32	*69.6*	0.482
Weaning (age in months) (mean; SD)	5.6	*1.0*	5.4	*1.5*	0.136
Caucasian (n; %)	32	*74.4*	36	*78.3*	0.670
Passive smoke (n; %)	9	*20.9*	7	*15.2*	0.585
Cough outside cold (n; %)	27	*63*	27	*59*	0.859
Sleep disorders (n; %)	37	*86*	37	*80*	0.576
Emergency events (n; %)	22	*51*	24	*52*	1.000
Hospitalization (n; %)	6	*14*	9	*20*	0.576
Missed school-days (n; %)	35	*81*	38	*83*	1.000
Responder (n, %)					
Mother	26	*60*	31	*67*	0.6729
Father	6	*14*	7	*15*	
Both	11	*26*	8	*17*	

^a^Chi-squared test, when condition were respected, or Fisher test was used to evaluate the association of categorical data between groups, Mann Whitney U test was used to compare quantitative not normally distributed variables between groups (Shapiro-Wilk test was used to assess normality of data)

^b^Sporadic missing values for a few variables examined

### Patients’ evaluation of the nebulizer and of the nasal aspirator

Over 90% of the participants in both groups considered of the DuoBaby nebulizer easy to use and clean (Table 2). Overall, 27 families initially had minor problems in implementing the nebulization, which were promptly solved by further consultation of the study nurse. Only 2 children did not tolerate the nebulization. The vast majority of the families reported an improvement of symptoms after the use of the nebulizer (80%, cases; 69%, controls), would use it again in the following winter season (70%, cases; 69%, controls) and would recommend the instrument to other families (77%, cases; 79%, controls) (Table 2).

**Table 2:** Answers to questionnaire on “DuoBaby” nebulizer among patients using the nebulizer equipped (cases; *n* = 43) or not equipped (controls; *n* = 46) with nasal aspirator

	Cases		Controls		
	*n* = 43^b^		*n* = 46^b^		p^a^
	n	*%*	n	*%*	
Handling the nebulizer					
Easy	39	*98*	39	*93*	0.241
Difficult	0	*0*	3	*7*	
Impossible	1	*3*	0	*0*	
Assembly of the nebulizer					
Easy	37	*95*	38	*90*	0.677
Quick solved problems	2	*5*	4	*10*	
Difficult	0	*0*	0	*0*	
Child’s tolerance of nebulization					
No problems	23	*58*	25	*60*	0.527
Some problems	17	*43*	15	*36*	
Inhalation impossible	0	*0*	2	*5*	
Cleaning of the nebulizer					
Easy	35	*90*	40	*95*	0.670
Quickly solved problems	3	*8*	1	*2*	
Difficult	1	*3*	1	*2*	
Nebulization improved symptoms					
Yes	18	*53*	17	*46*	0.726
Partially	13	*38*	17	*46*	
No	3	*9*	3	*8*	
Nebulization was beneficial					
Yes	30	*81*	27	*69*	0.035
Rather yes	1	*3*	7	*18*	
Rather no	3	*8*	5	*13*	
No	3	*8*	0	*0*	
Future use of nebulizer wished					
Yes	28	*70*	29	*69*	0.405
Rather yes	5	*13*	2	*5*	
Rather no	6	*15*	7	*17*	
No	1	*3*	4	*10*	
Nebulizer use recommendable to others					
Yes	31	*77.5*	34	*79*	0.386
Rather yes	5	*12.5*	4	*9*	
Rather no	2	*5.0*	5	*12*	
No	2	*5.0*	0	*0*	

Categorical data were summarized as numbers (n) and frequencies (%)

^a^Chi-squared test, when condition were respected, or Fisher test was used to evaluate the association of categorical data between groups

^b^Sporadic missing values for a few variables examined

Most participants considered the “DuoBaby” nasal aspirator easy to assemble and clean (Table 3). The removal of nasal secretions by aspiration was considered very or partially successful by 19 and 54% of the families, respectively. Similarly, the nasal symptoms were considered greatly or partially improved after aspiration by 17 and 46% of the parents, respectively. A positive impact of the use of the nasal aspirator was observed by about 50% of the parents on sleep, eating, and overall wellness of their children (Table 3). These values did not change when only parents of children younger or older than 24 months were examined (not shown).

**Table 3:** Parents’ opinion on the usability and efficacy of the DuoBaby’s nasal aspirator unit in 43 children with wheezing disorders

	n^a^	%
Assembly of DuoBaby Aspirator		
No problems	37	*93*
Quickly solved problems	1	*3*
Difficult	2	*5*
Aspirator cleaning		
No problems	33	*83*
Quickly solved problems	5	*12*
Difficult	2	*5*
Removal of nasal secretum		
Complete	7	*19*
Partial	20	*54*
Insufficient	10	*27*
Aspiration improved symptoms		
Yes	6	*17*
Partially	16	*46*
No	13	*37*
Aspiration improved sleep		
Yes	6	*17*
Rather yes	14	*40*
Rather no	7	*20*
No	8	*23*
Aspiration improved eating		
Yes	6	*17*
Rather yes	12	*33*
Rather no	8	*22*
No	10	*28*
Aspiration improved wellness		
Yes	10	*28*
Rather yes	10	*28*
Rather no	6	*17*
No	10	*28*
Aspiration was beneficial		
Yes	13	*34*
Rather yes	5	*13*
Rather no	8	*21*
No	12	*32*

^a^Sporadic missing values for two of the variable examined

### Frequency of respiratory symptoms and of salbutamol inhalation

**Fig. 2:**
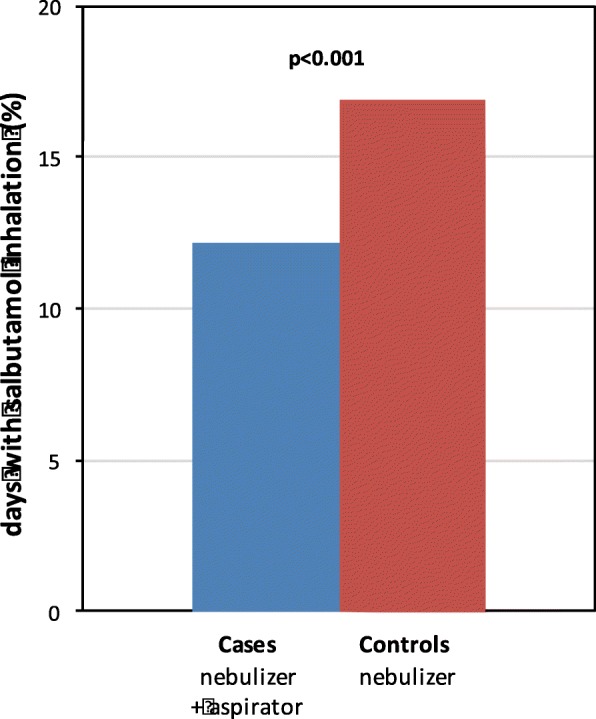
Salbutamol consumption (expressed in percentage of days) among patients using a DuoBaby nebulizer equipped (cases, *n* = 43) or not equipped (controls; *n* = 46) with an nasal aspirator. Percentage are calculated considering the total days with symptoms over the total day of reported days (see method for definition). Chi-squared test was used to evaluate frequency differences between independent groups

**Fig. 3:**
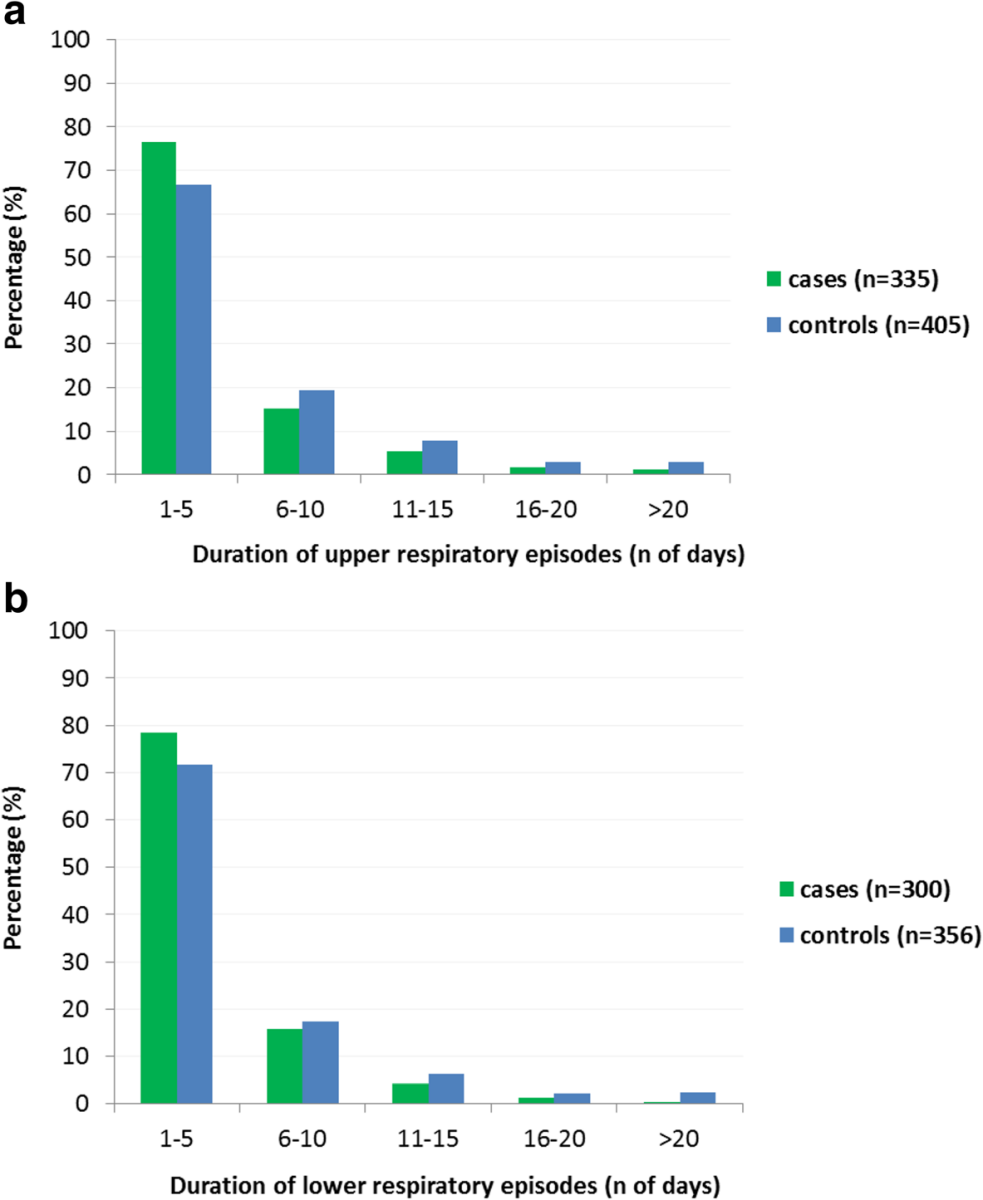
Frequency distribution of episode’s duration of **a** upper, and **b** lower respiratory symptoms (n of days) among patients using a DuoBaby nebulizer equipped (cases, *n* = 43) or not equipped (controls; *n* = 46) with an nasal aspirator

**Fig. 4:**
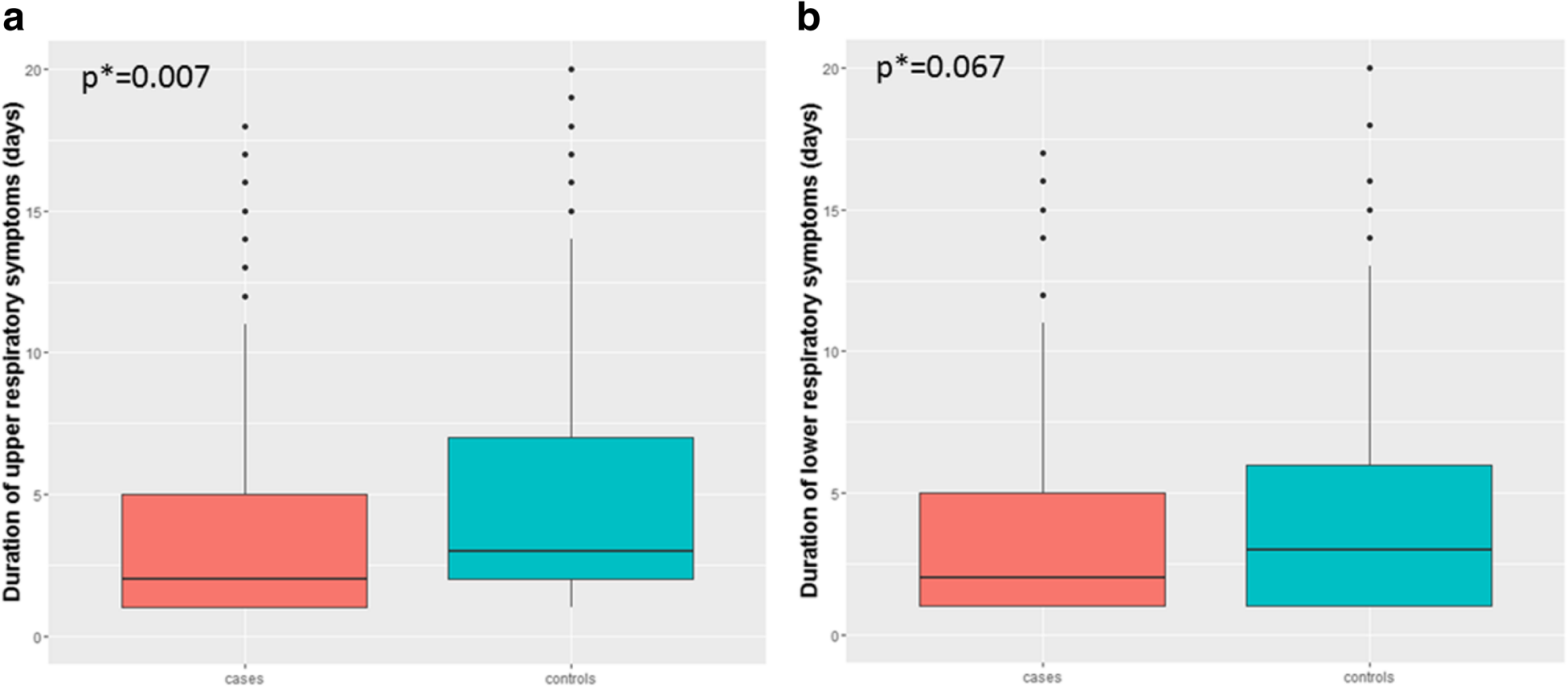
Difference in episode’s duration of **a** upper and **b** lower respiratory symptoms between patients using a DuoBaby nebulizer equipped (cases, *n* = 43) or not equipped (controls; *n* = 46) with an nasal aspirator. *P-value from mixed-effects Poisson regression

The mean (95% CI) number of days monitored after the T0 visit was of 102 (90–113) and 111 (102–120) in cases and controls, respectively. The patients using the nebulizer with nasal aspirator inhaled salbutamol less frequently than the patients using the nebulizer without nasal aspirator (12.2% vs 16.9% of the monitored days, *p* < 0.001) (Fig. 2). After stratification by age groups, this difference was observed only among the children older than 24 months (Additional file 1: Figure S2). The average percentage of days with any upper (25.0% vs 46.4%, *p* = 0.004) and with any lower (21.8% vs 32.8%, *p* = 0.022) airways symptom were both significantly lower in the group using the nebulizer with the nasal aspirator than in the group using the nebulizer without nasal aspirator (Table 4). In particular, a statistical significance was reached in the proportion of days with runny nose. A reduced frequency of productive cough among the cases vs controls (14.7% vs 20.3%, *p* < 0.033) was observed while no differences between the two groups were observed in the analysis of the frequency of wheezing (Table 4). The most relevant associations remained significant after stratification of the sample according to the use of controller therapy (Inhaled Corticosteroid, ICS) (Table 5) By contrast, after stratification for age, the observed differences in upper and lower airways symptoms were stronger and significant among children older than 24 months only (Additional file 1: Table S4). No difference was observed in the average number of episodes per month of upper [2.3 (95%CI: 1.8–2.8) vs 2.6 (95% CI:2.2–3.0), *p* = 0.122] or of lower [1.9 (95% CI:1.5–2.3) vs 2.1 (95% CI:1.7–2.4), *p* = 0.240] respiratory symptoms in the groups using the nebulizer with or without nasal aspirator (Fig. 3). In contrast, the duration of the episodes of upper [4.3 days (95% CI: 3.8–4.9) vs 5.7 days (95% CI: 5.0–6.4), *p* = 0.007] and of lower [3.8 days, (95% CI: 3.4–4.2) vs 4.4 days, (95% CI: 4.4–6.0), *p* = 0.067] respiratory symptoms were significantly or marginally shorter, respectively, in cases compared to controls (Fig. 4). When the cumulative number of days with symptoms was compared between the two groups, the cases had not only less days with upper and lower airways symptoms, but also less days with throat soreness, earache, and sleep disorders (Additional file 1: Figure S3).

**Table 4:** Frequency of symptoms among patients using a DuoBaby nebulizer equipped (cases, *n* = 43) or not equipped (controls; *n* = 46) with a nasal aspirator^a^

	Cases *n* = 43		Controls *n* = 46		p^b^
	Median^a^	IQR	Median^a^	IQR	
Nasal symptoms in the last 24 h	25.0	*(14.5–43.6)*	46.4	(27.4–58.4)	**0.004**
Runny nose	21.0	*(10.6–37.3)*	39.5	*(22.5–48.3)*	**0.021**
Stuffy nose	15.3	*(6.4–32.1)*	24.0	*(7.5–44.6)*	0.160
Mucus	14.4	*(3.4–34)*	21.1	*(7.1–38.3)*	0.289
Crusts	1.5	*(0–15.6)*	6.7	*(0–20.1)*	0.485
Bronchial symptoms in the last 24 h	21.8	*(14.5–37.3)*	32.8	(16.8–50.3)	**0.022**
Dry cough	12.7	*(3.5–25.8)*	17.8	*(5.8–32.1)*	0.229
Productive cough	14.7	*(6.5–18.9)*	20.3	*(9.3–37.7)*	**0.033**
Wheezing	3.6	*(0–10.3)*	2.6	*(0–10.8)*	1.000
Difficult breathing	0.0	*(0–3.3)*	0.0	*(0–3)*	0.836
Fever	3.1	*(0.8–5.3)*	4.2	*(1.6–6.9)*	0.152
Sore throat	1.4	*(0–3.2)*	0.7	*(0–4.3)*	0.800
Earache	0.0	*(0–1.2)*	0.0	*(0–2.2)*	0.105
Hoarseness	0.8	*(0–4.1)*	0.4	*(0–1.8)*	0.342
Sleep disorders	6.0	*(2.2–12)*	6.3	*(2.4–14.9)*	0.690
Loss of appetite	3.2	*(0–8.2)*	3.1	*(0.9–6.7)*	0.747
Anxiety / restlessness	2.0	*(0–4.7)*	1.0	*(0–6.6)*	0.537

^a^Median (IQR) of the percentages of days with symptoms calculated at individual level (see method for definition)

^b^Mann Whitney U test was used to compare quantitative not normally distributed variables between groups (Shapiro-Wilk test was used to assess normality of data)

Values marked in bold indicate statistically significant results with a *p* value < 0.05

**Table 5:** Frequency of symptoms among patients using a DuoBaby nebulizer equipped (cases, *n* = 43) or not equipped (controls; *n* = 46) with a nasal aspirator stratified for the use of controller therapy (Inhaled Corticosteroid, ICS)

	Cases				Controls					
	control therapy		no control therapy		control therapy		no control therapy			
	*n* = 6		*n* = 37		*n* = 10		*n* = 36		p^b^	p^c^
	Median^a^	IQR	Median^a^	IQR	Median^a^	IQR	Median^a^	IQR		
Nasal symptoms in the last 24 h	40.4	*(30.8–74.9)*	23.2	*(13.3–41.2)*	54.2	*(49.3–57.9)*	39.7	*(24.9–58.5)*	0.020	0.011
Bronchial symptoms in the last 24 h	0.0	*(0–0)*	0.0	*(0–0)*	*0.0*	(0–0)	0.0	*(0–0)*	0.035	0.031

^a^Median (IQR) of the percentages of days with symptoms calculated at individual level (see method for definition)

^b^p-value for cases vs controls from linear regression adjusted for control therapy

^c^Mann Whitney U test was used to compare quantitative not normally distributed variables between cases vs controls groups (eliminating subjects with control therapy)

No statistical differences in subjects with control therapy between cases vs controls

## Discussion

### Major findings

We completed a case-control study of 89 wheezing pre-school children in Germany, monitored for over 3 months during the cold season. To treat their child’s respiratory symptoms at home, we provided all parents with the same nebulizer, either equipped (cases) or not equipped (controls) with an automatic nasal aspirator. We observed a significantly lower burden of upper respiratory symptoms and cough as well as a lower use of nebulized salbutamol in cases compared to controls. The results of our pilot study suggest that a regular aspiration of the nasal cavities with an automatic device improves respiratory symptoms in wheezing children.

### Clinical impact of nasal aspiration

Our study shows an impact of nasal aspiration among both, children receiving or not receiving a regular controller therapy (inhaled corticosteroids, LTRA and/or nasal corticosteroids) on both, upper and lower airway symptoms: A) The episodes of upper airway symptoms were shorter but not less frequent among the cases than among controls. This observation suggests that the regular aspiration of the nose during colds may accelerate the resolution of a nasal viral infection but cannot prevent its recurrence. B) The reduced burden of cough suggests that a regular nasal aspiration during colds may prevent the post-nasal drip (the transition of nasal mucus in the pharynx), i.e. a well-known trigger of cough [14].

The postnasal drip is likely to be the most important factor linking the nasal aspiration to an improvement of respiratory symptoms. A pharmacological treatment of upper airway inflammation may improve bronchial hyper-responsiveness and cough [15–19]. Our results suggest that - especially in children older than 24 months - even the simple mechanical removal of nasal mucus may improve symptoms of the lower airways and reduce the need of salbutamol. A strong relationship between the upper and lower airways has been repeatedly shown in the literature [20–22].

It is more difficult to explain why a reduced use of salbutamol among cases was not paralleled by a reduced frequency of wheezing or difficult breathing. We can only speculate that parents had difficulties in the recognition of wheezing, a problem that has been previously reported in many studies [14, 23, 24]. On the other hand, the reduced frequency of cough – an asthma-like symptom - may have induced the parents to administer less salbutamol to their children.

### Parental opinion on nasal aspiration

Most parents perceived the handling, assembly and use of both, the nebulizer and the nasal aspirator unit of the DuoBaby device as easy and user friendly. Also the impact of the salbutamol inhalation was considered useful and highly effective on the symptoms of their child by most parents. About three quarters of the cases’ parents evaluated the removal of nasal secretion as totally or partially successful and about one third considered the use of the automatic nasal aspirator not beneficial for the respiratory symptoms of their children. This result is not surprising, given the wide age range and distribution of the study population, composed of infants, toddlers and pre-school children.

### Strengths and limitations

The idea that cleaning the nose is essential for a better respiratory health in children is a widely accepted concept for more than a century in westernized countries. However, to our knowledge, our study is the first exploring in a systematic way the influence of nasal aspiration on the occurrence of symptoms of the upper and lower respiratory airways. In addition, ours is one of the first studies taking advantage of an electronic diary allowing easy monitoring of the patients’ symptoms and medication use at home during a long observational period. The high adherence to the BreathMonitor e-Diary compilation and the good acceptance of this diagnostic procedure will be thoroughly discussed elsewhere. However, we must acknowledge some limitations of our study design. First, the use of the nasal aspirator was prescribed “as needed”. Therefore, within the group of cases, the patients using more often the nasal aspirator were also those with more severe nasal and bronchial symptoms. This bias prevents the investigation of a causal-effect link between the use of the nasal aspirator and the reduced frequency of respiratory symptoms of cases, compared to controls. Second, the study population, powered to answer the principal research question, is not large enough to ascertain which subgroup of patients better responded during the monitoring to the use of the nasal aspirator. Third, due to the nature of the study, the control group was not treated with a “placebo”-aspirator; moreover, the data on primary outcomes during the monitoring period were self-reported. Thus we cannot exclude a “placebo” effect due to the availability of the aspirator.

## Conclusions

Our study is the first suggesting that aspiration of the nasal secretions with an automatic device improves respiratory symptoms in wheezing children during the cold season in a middle European country. The study also shows a wide acceptance and efficacy of a nebulizer combined with a nasal aspirator in early childhood. Further studies are needed to identify the phenotype of URI and LRI best profiting of a combined nasal aspiration plus nebulization intervention. The outcome of these future trials will also answer whether this novel approach deserves to be considered in guidelines for the treatment of respiratory diseases in childhood.

## Additional file

Additional file 1: **Figure S1.** The DuoBaby nebulizer (a) and its functional scheme (b). **Figure S2.** Salbutamol consumption (expressed in percentage of days) among patients younger (cases *n* = 16, controls *n* = 16) or older (cases *n* = 27, controls *n* = 30) than 24 months and using a DuoBaby nebulizer equipped (cases) or not equipped (controls) with a nasal aspirator. Percentages are calculated considering the total days with symptoms over the total day of reported days (see method for definition). Chi-squared test was used to evaluate frequency differences between independent groups. **Figure S3.** Percentage of days with symptoms among patients using a DuoBaby nebulizer equipped (cases, *n* = 43) or not equipped (controls; *n* = 46) with an nasal aspirator. Percentage are calculated reporting the total days with symptoms on the total number of reported days. Chi-squared test was used to evaluate the association of categorical data between independent groups. Significant differences are highlighted as follows: *p < 0.05,** < 0.01, *** < 0.001. †Statistical significant differences after adjusting for multiple repeated measures through mixed-effects logistic regression. **Table S1.** List of the questions in the BreathMonitor APP (electronic Diary). **Table S2.** Questionnaire on the DuoBaby’s nebulizer unit. **Table S3.** Questionnaire on the use of the DuoBaby’s nasal aspirator. **Table S4.** Frequency of symptoms among patients using a DuoBaby nebulizer equipped (cases, *n* = 43) or not equipped (controls; *n* = 46) with a nasal aspirator stratified by age in months.* (DOCX 579 kb)

## Abbreviations

CI: Confidence interval
IQR: Interquartile range
LRI: Lower respiratory infections
LTRA: Leukotrienes Receptor Antagonist
MDI: Metered dose inhalers;
SD: Standard deviation;
UAD: United airway disease;
URI: Upper respiratory infections
VAS: Visual analogue scale
