# Correction to: The impact of nasal aspiration with an automatic device on upper and lower respiratory symptoms in wheezing children: a pilot case-control study

**DOI:** 10.1186/s13052-018-0518-5

**Published:** 2018-07-10

**Authors:** Antonio Pizzulli, Serena Perna, Anja Bennewiz, Holger Roeblitz, Salvatore Tripodi, Jakob Florack, Petra Wagner, Stephanie Hofmaier, Paolo Maria Matricardi

**Affiliations:** 1 Practice for Pediatric Allergy and Pneumology, Berlin, Germany; 2 Department of Pediatric Pulmonology, Immunology and Intensive Care Medicine, Charité - University Medicine Berlin, Augustenburger Platz, 1, 13353 Berlin, Germany; 3 Practice for General Pediatrics and Pediatric Cardiology, Berlin, Germany; 4 Practice for General Pediatrics and Pediatric Allergy, Berlin, Germany; 5 Pediatric Department and Pediatric Allergology Unit, Sandro Pertini Hospital, Rome, Italy

## Correction

The original article [1] contained a typesetting error in Table 5; this has now been corrected*.*

## References

[1] Pizzulli A (2018). The impact of nasal aspiration with an automatic device on upper and lower respiratory symptoms in wheezing children: a pilot case-control study. Ital J Pediatr.

