# Supplementary material for: The impact of nasal aspiration with an automatic device on upper and lower respiratory symptoms in wheezing children: a pilot case-control study
Source: Ital J Pediatr. 2018 Jun 14;44:68. doi: 10.1186/s13052-018-0489-6 (PMC6001015; doi:10.1186/s13052-018-0489-6)
Supplement: Supplementary file 1 — Figure S1. The DuoBaby nebulizer (a) and its functional scheme (b). Figure S2. Salbutamol consumption (expressed in percentage of days) among patients younger (cases n = 16, controls n = 16) or older (cases n = 27, controls n = 30) than 24 months and using a DuoBaby nebulizer equipped (cases) or not equipped (controls) with a nasal aspirator. Percentages are calculated considering the total days with symptoms over the total day of reported days (see method for definition). Chi-squared test was used to evaluate frequency differences between independent groups. Figure S3. Percentage of days with symptoms among patients using a DuoBaby nebulizer equipped (cases, n = 43) or not equipped (controls; n = 46) with an nasal aspirator. Percentage are calculated reporting the total days with symptoms on the total number of reported days. Chi-squared test was used to evaluate the association of categorical data between independent groups. Significant differences are highlighted as follows: *p < 0.05,** < 0.01, *** < 0.001. †Statistical significant differences after adjusting for multiple repeated measures through mixed-effects logistic regression. Table S1. List of the questions in the BreathMonitor APP (electronic Diary). Table S2. Questionnaire on the DuoBaby’s nebulizer unit. Table S3. Questionnaire on the use of the DuoBaby’s nasal aspirator. Table S4. Frequency of symptoms among patients using a DuoBaby nebulizer equipped (cases, n = 43) or not equipped (controls; n = 46) with a nasal aspirator stratified by age in months.* (DOCX 579 kb) [file 13052_2018_489_MOESM1_ESM.docx]

**ELECTRONIC REPOSITORY**

**The impact of nasal aspiration with an automatic device on upper and lower respiratory symptoms in wheezing children: A pilot case-control study.**

Antonio Pizzulli^1^, MD, Serena Perna^2^, M.Sc., Anja Bennewitz^3^ , MD, Holger Roeblitz^4^, MD, Salvatore Tripodi^5^, MD, Jakob Florack^2^, MD, Petra Wagner^2^, Stephanie Hofmaier^2^, MD, Paolo Maria Matricardi^2*^, MD

^1^ Practice for Pediatric Allergy and Pneumology, Berlin, Germany

^2^ Department of Paediatric Pneumology & Immunology, Charité - Universitätsmedizin Berlin, Berlin, Germany

^3^ Practice for General Pediatrics and Pediatric Cardiology , Berlin, Germany

^4^ Practice for General Pediatrics and Pediatric Allergy, Berlin, Germany

^5^ Pediatric Dept and Pediatric Allergology Unit, Sandro Pertini Hospital, Rome, Italy

**Tables:** 4

**Figures:** 3

**^*^Corresponding author:**

Paolo M. Matricardi

Dept. of Pediatric Pneumology and Immunology

Charité Medical University

Augustenburger Platz, 1

13353 Berlin, Germany

+49 30 450 566 406

+49 30 450 566 931

[paolo.matricardi@charite.de](mailto:paolo.matricardi@charite.de)

**Figure S1** The DuoBaby nebulizer (a) and its functional scheme (b).

**Figure S2** Salbutamol consumption (expressed in percentage of days) among patients younger (cases n=16, controls n=16) or older (cases n=27, controls n=30) than 24 months and using a DuoBaby nebulizer equipped (cases) or not equipped (controls) with a nasal aspirator. Percentages are calculated considering the total days with symptoms over the total day of reported days (see method for definition). Chi-squared test was used to evaluate frequency differences between independent groups.

**Figure S3** Percentage of days with symptoms among patients using a DuoBaby nebulizer equipped (cases, n=43) or not equipped (controls; n=46) with an nasal aspirator. Percentage are calculated reporting the total days with symptoms on the total number of reported days. Chi-squared test was used to evaluate the association of categorical data between independent groups. Significant differences are highlighted as follows: *p<0.05,**<0.01, ***<0.001. †Statistical significant differences after adjusting for multiple repeated measures through mixed-effects logistic regression.


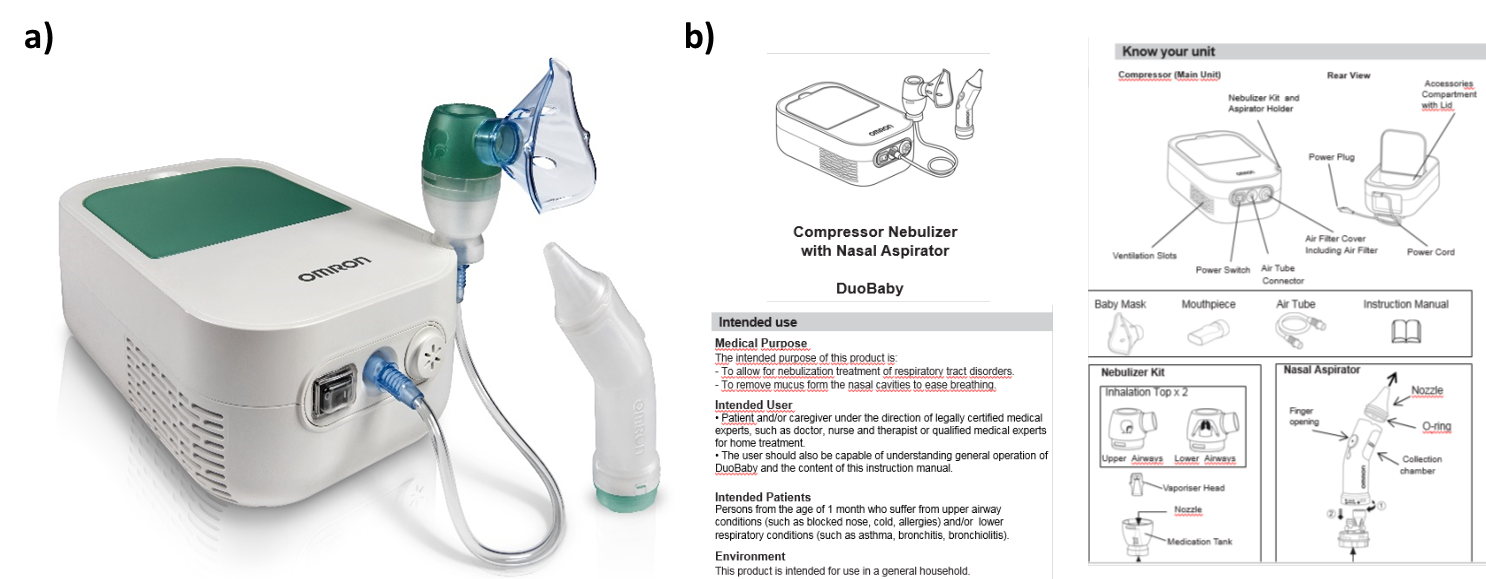
**Figure S1**

**Figure S2**

**
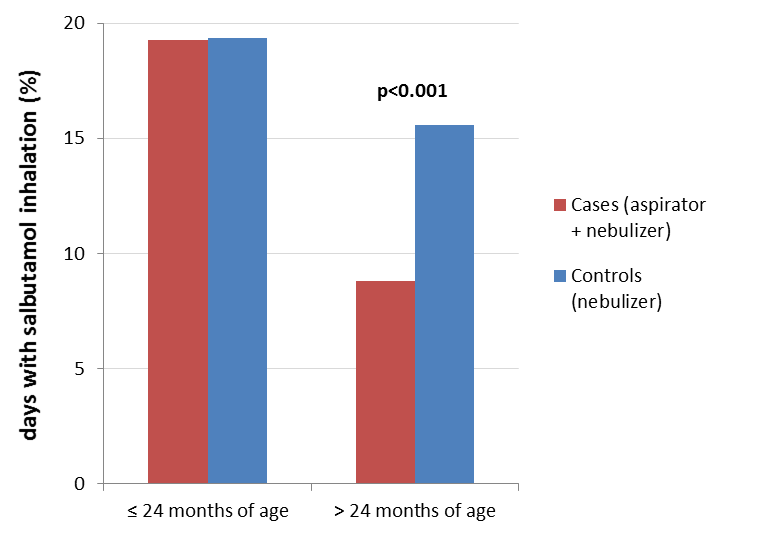
**

**Figure S3**

**
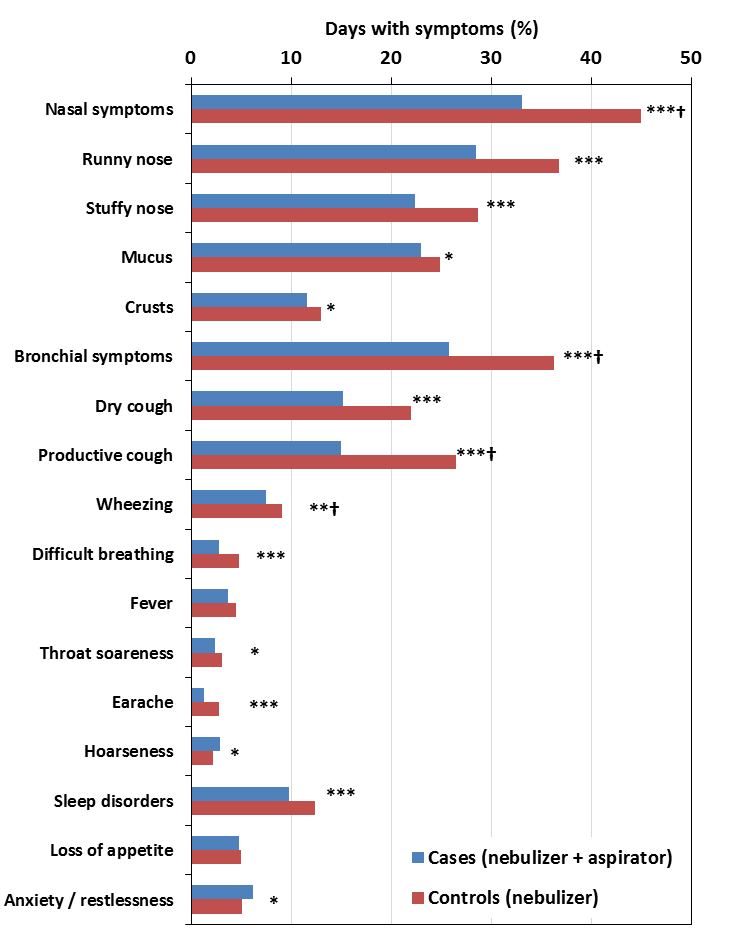
**

| **Table S1** - List of the questions in the BreathMonitor APP (electronic Diary). | | | | | |
| --- | --- | --- | --- | --- | --- |
|  | **Question** | | | | **Answers** |
|  |  |  | | |  |
| **1** | **Did your child have nasal symptoms in the last 24 hours?** | | | | Y/N |
|  | **1a** | | | Runny nose | 0-3* |
|  | **1b** | | | Stuffy nose | 0-3 |
|  | **1c** | | | Yellow mucus | 0-3 |
|  | **1d** | | | Green mucus | 0-3 |
|  | **1e** | | | Clear mucus | 0-3 |
|  | **1f** | | | Crusts | 0-3 |
| **2** | **Did your child have other respiratory symptoms last night?** | | | | Y/N |
|  | **2a** | | | Dry cough | 0-3 |
|  | **2b** | | | Productive cough | 0-3 |
|  | **2c** | | | Whistling breathing | 0-3 |
|  | **2d** | | | Difficulty in breathing | 0-3 |
| **3** | **Did your child use the Duo Baby today?** | | | | Y/N |
|  | **3a** | | | Did you aspirate your kid's nose today? If yes, how often:† | 0-3+ |
|  | **3b** | | | Did your child inhale saline today? If yes, how often: † | 0-3+ |
|  | **3c** | | | Has your child inhaled salbutamol today? If yes, how often:† | 0-3+ |
| **4** | **Did your child take other medications?** | | | | Y/N |
|  | **4a** | | Paracetamol? | | Y/N |
|  | **4b** | | Ibuprofen? | | Y/N |
|  | **4c** | | Nasal drops containing drugs? | | Y/N |
|  | **4d** | | Non-medicinal nasal drops? | | Y/N |
|  | **4e** | | Anti-cough syrup? | | Y/N |
|  | **4f** | | Anti-histamines? | | Y/N |
|  | **4g** | | Other drugs by inhalation? | | Y/N |
|  | **4h** | | Any others medications? | | Y/N |
| **5** | **Did you consult a doctor because of the child's symptoms?** | | | | Y/N |
|  | **5a** | | Did you contact your family doctor? | | Y/N |
|  | **5b** | | Did you go to the emergency department? | | Y/N |
| **6** | **In the last 24 hours, had your child:** | | | |  |
|  | **6a** | | fever(>38°)? | | Y/N |
|  | **6b** | | sore throat | | Y/N |
|  | **6c** | | earache | | Y/N |
|  | **6d** | | hoarseness | | Y/N |
|  | **6e** | | sleep disorders | | Y/N |
|  | **6f** | | loss of appetite | | Y/N |
|  | **6g** | | anxiety/restlessness | | Y/N |
|  | **6h** | | moodiness | | Y/N |
|  |  | |  | |  |
| *0=none, 1=mild, 2=moderate; 3=severe | | | | |  |
| † 0=none, 1= one time, 2=two times, 3+= 3 or more times | | | | |  |

| **Table S2 -** Questionnaire on the DuoBaby's nebulizer unit |
| --- |
|  |
| 1. How was the handling of the "DuoBaby" device? |
|  |
| 2. How was the assembly of the "DuoBaby" nebulizer? |
|  |
| 4. How did your child tolerate the inhalation? |
|  |
| 5. How was the cleaning of the nebulizer? |
|  |
| 6. Did the child's inhalation symptoms improve? |
|  |
| 7. Do you think your child had some benefit from inhalation? |
|  |
| 8. Are you interested in a future use of the device? |
|  |
| 9. Would you recommend others the use of the nebulizer? |
|  |

| **Table S3 -** Questionnaire on the use of the DuoBaby's nasal aspirator |
| --- |
|  |
| 1. How was the construction of the "DuoBaby" aspirator? |
|  |
| 2. How was the cleaning of the aspirator? |
|  |
| 3. How successful was the removal of nasal secretum with the aspirator? |
|  |
| 4. Did the child's respiratory symptoms improve after the aspiration? |
|  |
| 5. Was your child able to sleep better after aspiration? |
|  |
| 6. Was your child able to eat or drink better after the aspiration? |
|  |
| 7. Did you notice any improvement in your child's wellness after the aspiration? |
|  |
| 8. Do you think your child had some benefit from aspiration? |
|  |
